# On-chip constructive cell-network study (II): on-chip quasi-*in vivo *cardiac toxicity assay for ventricular tachycardia/fibrillation measurement using ring-shaped closed circuit microelectrode with lined-up cardiomyocyte cell network

**DOI:** 10.1186/1477-3155-9-39

**Published:** 2011-09-19

**Authors:** Fumimasa Nomura, Tomoyuki Kaneko, Akihiro Hattori, Kenji Yasuda

**Affiliations:** 1 Department of Biomedical Information, Division of Biosystems, Institute of Biomaterials and Bioengineering, Tokyo Medical and Dental University, 2-3-10 Kanda-Surugadai, Chiyoda, Tokyo 101-0062, Japan

## Abstract

**Backgrounds:**

Conventional *in vitro *approach using human ether-a-go-go related gene (hERG) assay has been considered worldwide as the first screening assay for cardiac repolarization safety. However, it does not always oredict the potential QT prolongation risk or pro-arrhythmic risk correctly. For adaptable preclinical strategiesto evaluate global cardiac safety, an on-chip quasi-*in vivo *cardiac toxicity assay for lethal arrhythmia (ventricular tachyarrhythmia) measurement using ring-shaped closed circuit microelectrode chip has been developed.

**Results:**

The ventricular electrocardiogram (ECG)-like field potential data, which includes both the repolarization and the conductance abnormality, was acquired from the self-convolutied extracellular field potentials (FPs) of a lined-up cardiomyocyte network on a circle-shaped microelectrode in an agarose microchamber. When Astemisol applied to the closed-loop cardiomyocyte network, self-convoluted FP profile of normal beating changed into an early afterdepolarization (EAD) like waveform, and then showed ventricular tachyarrhythmias and ventricular fibrilations (VT/Vf). QT-prolongation-like self-convoluted FP duration prolongation and its fluctuation increase was also observed according to the increase of Astemizole concentration.

**Conclusions:**

The results indicate that the convoluted FPs of the quasi*-in vivo *cell network assay includes both of the repolarization data and the conductance abnormality of cardiomyocyte networks has the strong potential to prediction lethal arrhythmia.

## Findings

Lethal arrhythmia has been one of the major safety concerns for the pharmaceutical industry in selecting and developing compounds. Hence, effects of compounds on the cardiovascular system like blood pressure, heart rate, and electrocardiogram should be assessed appropriately [1,2]. Integrated assay systems using human ether-a-go-go related gene (hERG)-transfected HEK-293/CHO-cells (hERG assay), isolated animal tissues (APD or MAPD assay) and conscious and/or anesthetized whole animals (QT or MAPD assay), are currently used to identify QT prolongation [3-5]. Those assay systems are useful to predict QT prolongation risk (inhibition of repolarization process) and conductance's abnormalities. However, they cannot fully predict the potential pro-arrhythmic activities such as Torsades de Pointes (TdP), ventricular tachycardia (VT) or ventricular fibrillation (Vf) induced by compounds [6-8]. In this context, there is a longstanding and urgent need for a surrogate marker that can distinguish the torsadogenic potential from the QT interval duration.

We here propose a quasi-in vivo cardiac toxicity assay, which is a new in-vitro cell network assay technology platform where on-chip technology is used as an assay tool to bridge the gap between conventional *in vitro *single-cell-based studies and *in vivo *human clinical settings in terms of cardiac toxicity of new chemical entities for drug development. Potential advantages of the proposed strategy of our quasi-in vivo assay to predict lethal arrhythmia (TdP/VT/Vf) by evaluation of spatial cell-to-cell conductance fluctuation using the on-chip cell network loop which can choose different conductance pathways of human cardiomyocytes among neighboring circulations. We have shown that the on-chip cell network loop model would offer the novel platform to assess the proarrhythmic (not only TdP but also VT/Vf) risks of compounds.

**Figure 1:**
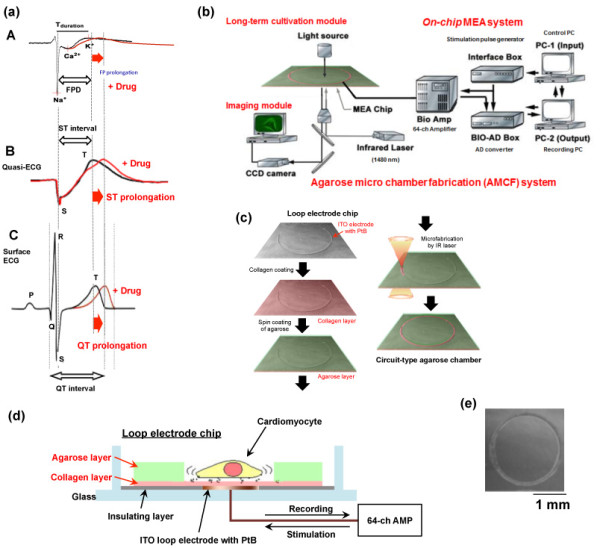
**On-chip quasi-in vivo cardiac toxicity measurement assay fabrication**. (a) Relationship of field potential profile (FP) of single cardiomyocyte (A), quasi -*in vivo *ECG signals convoluted from FP profiles of lined-up cardiomyocyte network (B), and the surface electrocardiogram (ECG) of individuals (C). (b) System set-up. (c) Fabrication procedure of closed circuit-shaped lined-up cardiomyocyte network. (d) Crossectional view of loop electrode chip. (e) Phase-contrast image of the ring-shaped closed circuit electrodes. Bar, 1 mm.

Figure 1 shows the principle and the system set-up of the on-chip quasi-*in vivo *cell network measurement system. Figure 1(a) shows the relationship of electrophysiological profiles of the single cardiomyocyte extracellular field potential (FP) profile (A), the convoluted FP profiles of lined-up cardiomyocyte network (B), and the surface electrocardiogram (ECG) of individuals (i.e., *in vivo *surface ECG) (C). The surface ECG is a transthoracic electrical signal of the heart muscle depolarizes during each heart beat externally recorded by skin electrodes. A typical ECG tracing of the cardiac cycle (heartbeat) consists of a P wave, a QRS complex, and a T wave. ST interval in quasi-ECG model is considered to correspond the duration of S wave to the apex of T wave in surface ECG. Hence the convolution of propagating FP signals in lined-up ventricles cardiomyocyte cell network (B) should represent the characteristics of a piece of ventricles tissue, i.e., quasi-*in vivo *ECG signals in ventriucles (ST interval).

As shown in Figure 1(b), the agarose microchamber fabrication system (AMCF) was used for quasi-*in vivo *preclinical cardiac toxicity assay, in which extracellular signals (FP) of cardiomyocyte cells in geometrically patterning chambers have been recorded with an multielectrode array (MEA) system [9-13]. For the on-chip quasi-*in vivo *measurement of quasi-*in vivo *ECG signals, a closed circuit-shaped lined-up cardiomyocyte network was cultivated in a closed circuit-shaped agarose microchamber fitting to the closed loop single electrode, which was fabricated by a spot-heating of a portion of agarose layer as follows (Figure 1(c)): The chip was first coated with collagen Type I-C (Nitta gelatin), and then were spin-coated with 2% (w/v) agarose (GenePure LowMelt ISC BioExpress) at 1500 rpm for 20 s. To form the closed circuit chamber for the ring-shaped cell network model, a portion of agarose gel layer on the surface of MEA was removed by the spot heating of focused 1480 nm infrared laser. Figure 1(d) also shows the cross-sectional schematic drawing of the spatial arrangement of cardiomyocytes in a closed loop-shaped indium tin oxide (ITO) electrode in the quasi-*in vivo *chip.

Cardiomyocytes were isolated from 13-day-old mouse embryos (ICR) and were placed in agarose micro-chamber on the MEA chip with a concentration having 5 ×10 ^5 ^cells/ml (more than 73.6% purity of beating cardiomyocytes), and cultivated in a cell culture medium (Invitrogen DMEM supplemented with 10% fetal bovine serum, 100 U/ml penicillin, and 100 mg/mL streptomycin) at 37°C with a humidified atmosphere of 95% air and 5% CO_2_. The attached cells on the collagen layer in the agarose micorochamber grew and extended to form the electrophysiologically connected cardiomyocyte within 7 days (Figure 1(e)).

**Figure 2:**
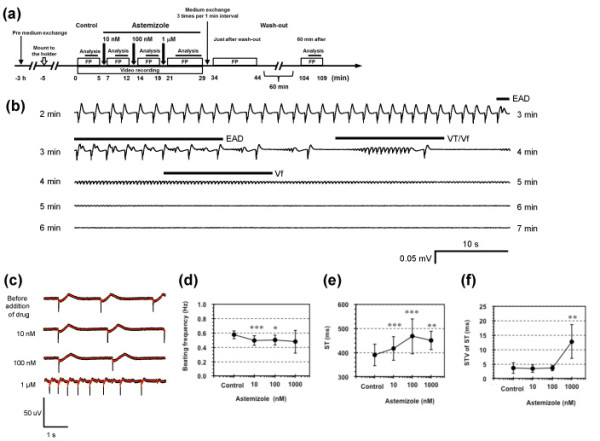
**Abnormal beating and fibrillation caused by arrhythmia compound (Astemizole) in a circuit-type *mouse *embryonic cardiomyocyte network**. (a) Procedure of compound application. (b) time course FP (quasi-*in vivo *ECG of ST interval) profiles after addition of 1 μM Astemizole, (c) Astemizole concentration dependence of FP waveform change. (d)-(f) Astemizole concentration dependence of FP waveform change in beating frequency (d), ST (e), and short-term variability (STV) of ST (f) in three different ring shape electrodes of 1 mm in diameter. *: p < 0.05, **: p < 0.01, ***: P < 0.005, compareed to control in paired t-test.

Figure 2 shows the results of quasi-*in vivo *ECG signals acquired from the closed loop-shaped circuits. To evaluate the prediction ability of ventricular lethal arrhythmia using this cell network assay, Astemizole, which is one of the false-negative compounds on APD prolongation in guinear-pig papillary muscle assay, was applied to the cardiomyocyte networks for VT/Vf measurement with the procedures as shown in Figure 2(a). In Figure 2(b), the time course of FP (quasi-*in vivo *ECG) waveform changed from a normal beating to an early afterdepolarization (EAD) like waveform, which maintains their beating intervals with abnormal additional depolarization during phase 2 or phase 3 of the cardiac action potential before normal repolarization is competed, and then changed into VT/Vf about 3 min after 1 μM Astemizole application. It should be noted that the EAD and ST interval prolongation started simultaneously in this example. Figure 2(c) shows the example of the FP (quasi-*in vivo *ECG) waveform of ring-shaped cardiomyocyte network in the short closed loop electrodes (1 mm in diameter) and we confirmed that the length of ST interval apparently prolonged and the fluctuation (short-term variability: STV [14]) of ST duration increased according to the increase of Astemizole concentration.

Figures 2(d) to 2(f), and Table 1 show the summaries of the ring-shaped circuits (1 mm in diameter), indicating beating frequencies (d), ST (e), and STV of ST (f). As shown in the above data, the acquired signals showed ST prolongation and STV increase independent to the circuit diameter differences and similar to the surface ECG signals, but also the occurrence of EAD or subsequent VT/Vf like waveforms, which is similar to the results of *in vivo *QT screening [15].

**Table 1:** Parameter of field potential recordings of cardiomyocytes circuit on ring-type electrodes at the administration of Astemizole

Sample	Beating frequency					ST							
	
	Mean ± SD (Hz)					Mean ± SD (ms)				STV (ms)			
	
	Before	10 nM	100 nM	**1 μM **^**†**^		Before	10 nM	100 nM	**1 μM **^**†**^	Before	10 nM	100 nM	**1 μM **^**†**^
1	0.53 ± 0.04	0.47 ± 0.06	0.54 ± 0.06	0.29 ± 0.09	^a^	408 ± 10	427 ± 9	509 ± 23	446 ± 12	7.8	6.1	4.6	9.2
2	0.5 ± 0.05	0.45 ± 0.05	0.47 ± 0.05	0.68 ± 0.26	^a^	403 ± 5	425 ± 7	481 ± 12	473 ± 49	3.4	3.1	3.3	16.9
3	0.63 ± 0.09	0.54 ± 0.05	0.52 ± 0.08	0.47 ± 0.13	^a^	364 ± 6	386 ± 5	420 ± 7	441 ± 27	3.0	2.7	3.6	7.7
4	0.65 ± 0.06	0.59 ± 0.05	0.56 ± 0.05	0.41 ± 0.09	^a^	445 ± 9	491 ± 14	580 ± 27	423 ± 26	2.9	3.5	4.6	21.7
5	0.54 ± 0.11	0.38 ± 0.1	0.36 ± 0.06	0.28 ± 0.12	^b^	311 ± 6	337 ± 4	356 ± 4	410 ± 36	1.9	1.7	2.2	5.9
6	0.58 ± 0.06	0.52 ± 0.04	0.52 ± 0.06	0.58 ± 0.22	^a^	425 ± 7	455 ± 10	497 ± 7	526 ± 19	3.6	3.6	4.0	11.0
7	0.53 ± 0.04	0.47 ± 0.06	0.54 ± 0.06	0.29 ± 0.09	^a^	371 ± 8	402 ± 6	428 ± 4	435 ± 54	3.0	3.0	2.6	17.1

Mean	0.57	0.49	0.50	0.48		390	418	467	451	3.7	3.4	3.5	12.8
SD	0.05	0.07	0.07	0.16		45	49	73	38	1.9	1.3	0.9	5.8
t-tset		***	*				***	***	**				**

a: Fibrillation after abnormal beating, b: Abnormal beating, †: Calculation from FP recordings just before abnormal beating.

*: p < 0.05, **: p < 0.01, ***: P < 0.005, compareed to control in paired t-test.

There are several advantages of this ring-shaped cell network: First, the round shape is more strong and stable to maintain their spatial cell network arrangements than the linearly lined-up cardiomyocyte network. For example, when we cultivate cardiomyocytes in the linearly lined-up network, the cardiomyocytes at the both ends of linearly lined-up cell network was easily shrunk to the center during their beating because of their force generation and detachment from substrate. Next, as there is no end of cell network in closed loop design, there is no need to arrange pacemaker cardiomyocytes to particular points such as the end of cell network in lined-up model. Third and finally, there is a potential to become a virtual re-entry model, in which the generation of irregular propagation of excitable conduction should be enhanced during its circulation.

The above results also shows the potential of the next generation of on-chip *in vitro *screening assay using cell network measurements of repolarization and conductance abnormalities for the estimation of delayed repolarization-associated ventricular tachyarrhythmia (e.g., Torsade de Pointes, VT, Vf), which could not be acquired from the *in vivo *assay such as hERG and APD measurements.

A simple quasi-*in vivo *ECG measurement assay using closed loop electrode cardiomyocyte network has been developed and the results showed the typical arrhythmia profiles, in which both the temporal repolarization information and the spatial beating propagation information appeared.

## Competing interests

The authors declare that they have no competing interests.

## Authors' contributions

FN, TK and AH carried out whole experiments and participated in the design of the study and contributed to the drafting of the manuscript. KY conceived of the study, participated in its design and coordination and drafted the manuscript. All authors read and approved the final manuscript.
